# Burdens of infection control on healthcare workers: a scoping review

**DOI:** 10.1016/j.jhin.2023.12.003

**Published:** 2023-12-21

**Authors:** R. Ungar, R. Gur-Arie, G.S. Heriot, E. Jamrozik

**Affiliations:** a Melbourne Medical School, Faculty of Medicine, Dentistry and Health Sciences, The University of Melbourne, Melbourne, Australia; b Center for Health Promotion and Disease Prevention, Edson College of Nursing and Health Innovation, Arizona State University, Phoenix, AZ, USA; c Department of Infectious Diseases, University of Melbourne, Peter Doherty Institute for Infection and Immunity, Melbourne, Australia; d Ethox Centre and Pandemic Sciences Institute, University of Oxford, Oxford, UK; e Department of Medicine, Royal Melbourne Hospital, University of Melbourne, Melbourne, Australia; f Monash Bioethics Centre, Monash University, Melbourne, Australia

**Keywords:** Infection prevention, Quality, Guideline adherence, Healthcare attitudes

## Abstract

**Introduction:**

Hospital-acquired infections (HAIs) pose a significant risk to patients, and are a major focus of infection prevention and control policies (IPC). One under-recognized reason for the generally poor compliance with IPC is that it is burdensome for healthcare workers (HCWs).

**Aim:**

To identify the burdens of IPC for HCWs.

**Methods:**

PubMed and CINAHL were searched for studies published in English since 2000 regarding compliance with IPC and the burdens associated with compliance. After screening 1018 initial results, 25 articles were included in the final review.

**Results:**

Evidence was found for burdens including dermatological complications, headaches, sensory symptoms and time pressure. Tools designed to measure compliance with IPC have limitations, and rarely assess the burdens of compliance. A strong safety culture predicted positive compliance, while knowledge of the underlying rationale for IPC had a non-linear relationship with compliance.

**Conclusion:**

Future research should clarify IPC-related burdens and how these may be minimized to achieve better compliance.

## Introduction

**Figure 1:**
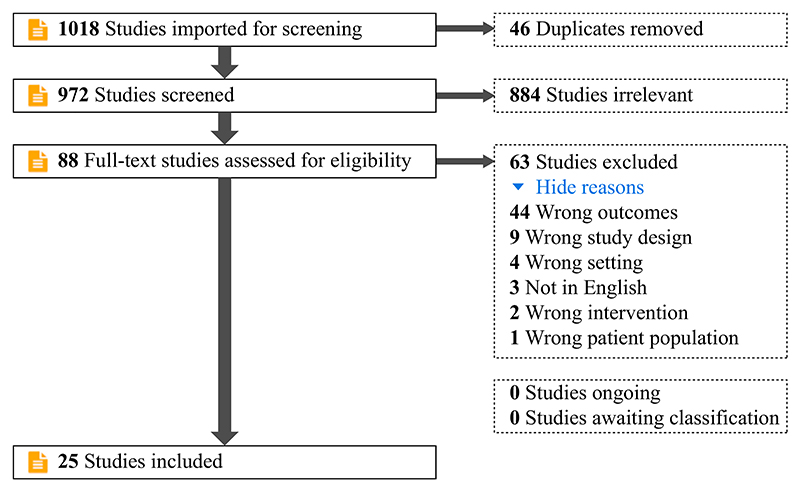
PRISMA diagram.

**Figure 2:**
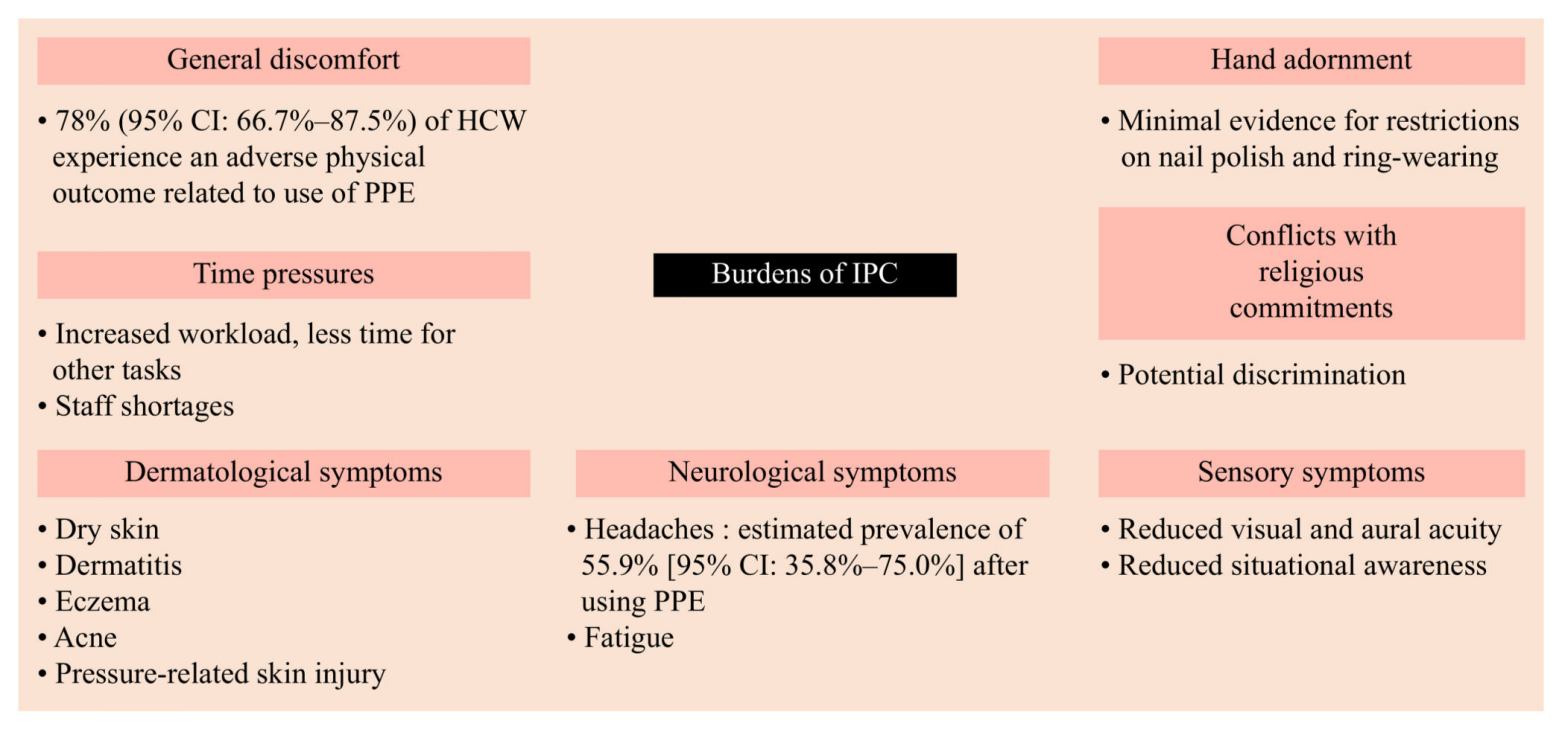
Visual representation of the taxonomy of burdens created by infection control and prevention policies. CI, confidence interval; HCW, healthcare workers; PPE, personal protective equipment.

Hospital-acquired infections (HAIs) impose costs on health systems, patients and staff. A large proportion of HAIs are preventable via compliance with appropriate infection and control policy/policies (IPC). Low adherence to IPC is a major obstacle to global efforts to reduce HAIs. In addition to the financial costs of IPC for health systems, there are personal costs borne largely by healthcare workers (HCWs), while the primary beneficiaries are patients. Many papers on infection control study HCW compliance with specific IPC, but rarely consider the cumulative burdens created by IPC on those workers. This paper aims to review the literature on HCW IPC compliance, and to categorize the burdens of compliance for HCWs. The paper concludes by highlighting areas for future research (Figures 1 and 2).

Patient safety is fundamental to health care [1]. However, interactions with a healthcare system pose significant risks. For example, in healthcare systems in high-income countries, 5−15% of patients, and up to 25% of patients in intensive care units, will develop an HAI. This figure doubles in low-income countries [2]. HAIs have major impacts on patient outcomes, healthcare costs, economies, and perceptions of health care. In Australia, approximately 165,000 people will contract an HAI each year [3]. However, 55−70% of all HAIs may be avoided by compliance with IPC [4]. Compliance with IPC is generally low, although measurement may be challenging. Using hand hygiene (HH) as a point estimate for IPC compliance, adherence has been estimated to range between 30% and 61% globally [5].

While IPC-related burdens may be one reason for low compliance, others include: (i) access to personal protective equipment (PPE) [6–8]; (ii) high workloads and perceptions of limited time [9–13]; (iii) institutional factors, such as poor supervision of junior staff [14]; (iv) a culture which does not emphasize safety [7,8,15–17]; and (v) a lack of appropriate, context-specific IPC education and training [18,19]. Measures aimed at improving compliance may address these factors and/or the burdens associated with IPC compliance. This review focused on barriers to compliance, and burdens for HCWs associated with IPC.

## Methods

PubMed and CINAHL were searched using the following keywords: (‘Infection Control’[Mesh] OR ‘Universal Pre-cautions’[Mesh]) AND (‘Personnel, Hospital’[Mesh] OR ‘Health Personnel’[Mesh]) AND (‘Guideline Adherence’[Mesh] OR ‘Quality of Health Care’[Mesh] OR ‘Health Knowledge, Attitudes, Practice’[Mesh] OR ‘burden’ OR ‘compliance’) (see Appendix 1 in the online supplementary material for full details of the search strategy). The search was limited to articles written in English and published between 1^st^ January 2000 and 16^th^ May 2022.

Consistent with a scoping review approach, this review aimed to capture a wide breadth of the literature in this evolving field, with assessment of quality at the time of selecting papers from the initial search. Where the first reviewer was uncertain regarding inclusion, papers were assessed by a second reviewer. Appendix 2 (see online supplementary material) details all studies included in this review.

Papers were excluded if they did not describe the burdens of complying with IPC, if they described scenarios which were not located in clinical spaces, or if they did not focus exclusively on HCWs. Conference abstracts were excluded due to lack of detail. The methodology was assessed for each paper included in this review, including appropriate population selection, randomization, blinding, defined primary and secondary outcome measures, and statistical analysis where relevant.

## Results

In total, 1018 articles were identified: 518 from CINHAL, 421 from PubMed, and the remainder from the authors’ personal files. After removing duplicates, the titles and abstracts of 972 articles were screened. Full-text analysis of 88 papers was then undertaken, yielding 25 papers for inclusion.

### Burdens

The reported burdens of IPC compliance among HCWs include: (i) general discomfort; (ii) dermatological symptoms; (iii) sensory symptoms; (iv) neurological symptoms; (v) privacy infringements; (vi) time pressures; (vii) conflicts with religious commitments; and (viii) limitations on hand adornment.

### General discomfort

High-quality evidence suggests that general discomfort is a barrier to the use of PPE [13,19,20]. A systematic review by Galanis *et al*. (2021) estimated that 78% [95% confidence interval (CI) 66.7−87.5%] of HCWs will experience at least one adverse physical outcome related to PPE [21], although the authors did not comment on links between specific types of PPE and specific adverse outcomes.

### Dermatological symptoms

Among dermatological burdens, dry skin was among the most commonly reported, with a pooled prevalence of 54.4% (95% CI 25.4−81.8%) among HCWs from eight studies [21]. Up to 85% of nurses reported dermatitis on their hands at some point in their career [5]. Handwashing, alcohol rub and use of gloves are all associated with higher rates of skin drying and irritation [5,21]. In particular, repeat exposure to HH agents with consecutive shifts is a risk factor for skin irritation [5,21]. Environmental cleaning agents were linked to dermatitis in HCWs by an audit of the Czech National Registry of Occupational Diseases, but the study was unable to determine the underlying disease incident rate [22]. Other occupational skin conditions include those arising from the use of face masks. Mask wearing is associated with development of contact dermatitis, pressure-related skin injury, acne and moisture-associated dermatitis [23].

### Sensory symptoms

The use of PPE may create vision and hearing impairments. Between 84% and 89% of HCWs using level 1 PPE while attending to the care of patients with coronavirus disease 2019 (COVID-19) reported reduced visual and aural acuity. Multi-variate analysis showed that full PPE was associated with impairment in spoken communication, reducing situational awareness [13].

### Neurological symptoms

Four papers published during the COVID-19 pandemic discussed neurological symptoms associated with PPE. Headaches were the most common adverse event among HCWs, with an estimated prevalence of 55.9% (95% CI 35.8−75.0%) after using PPE [21]. Another prospective survey of HCWs found that 44.98% of respondents developed a headache after donning PPE [24], with a medium intensity of 6/10 on a visual analogue scale. Although neither of these papers linked specific types of PPE to the development of headaches, other studies have linked N95 masks to headaches in HCWs [25]. Qualitative research, involving focus groups with 21 HCWs in India, echoed these findings [8].

### Time pressures

HCWs often navigate heavy workloads and time constraints, and these factors are associated with non-compliance with IPC [18,26]. A 2021 survey found that 23.9% of respondents cited ‘lack of time’ as a barrier to the use of PPE [6]. Use of multiple types of PPE requires a significant time investment, reducing time available for other tasks. A 2013 survey found that 35.2% of participants agreed ‘It is inconvenient/uncomfortable to use all the recommended PPE while doing patient care’ [27]. Compliance appears to be better in high-acuity settings where staff ratios are higher [19]. A 2020 review concluded that there was moderate evidence to suggest an increased workload was seen by HCWs as a barrier to adherence [20].

While time constraints may limit IPC compliance, so too, IPC compliance reduces time available for other tasks. On average, each patient in an intensive care unit is contacted directly 159 (95% CI 144−178) times per day and contacted indirectly 191 (95% CI 174−210) times per day. Given observed post-contact HH rates of 43% and 12% for direct and indirect contacts, respectively, 100% HH compliance among all HCWs would require an additional 171.4 min/patient/day [28]. Time constraints are sometimes exacerbated by reduced staffing ratios and staff shortages, and may affect patient outcomes [29].

### Conflicts with religious commitments

There is potential for discrimination in the design and implementation of IPC. For example, in discussion of a Swedish legal case about the IPC of ‘Bare Below the Elbow’ and whether a Muslim dental professional might wear disposable over-sleeves with a niqab in line with her religious beliefs, it was noted that ‘the Court decided that?. oversleeves, being solely for the benefit of the worker, did not fulfil a patient safety function’ [30]. The Court ruled against the dental professional, referring to potential risks to the patient created by the proposed adjustments.

### Hand adornment

IPC often direct HCWs to remove all hand adornment or limit jewellery to a single band ring and unchipped nail polish. This may represent a minor burden of personal freedoms of expression among staff, yet the benefits of such rules remain unclear [31,32]. While chipped nail polish may provide an uneven surface which could harbour micro-organisms, no trials have evaluated whether the use of nail polish by HCWs is associated with increased rates of surgical site infections [33]. Some low-quality studies have suggested that nail polish and ring-wearing reduce the quality of surgical scrubs and HH, but the extent to which this effect correlates with infection rates remains unclear [33]. Overall, directives to limit hand adornment are not based on strong evidence [31].

### Barriers to measuring compliance

In their 2014 review, Valim *et al*. identified 18 unique instruments designed to assess IPC compliance. It found ‘Health professionals worldwide appear to be selective when following SP [standard precautions], and the same can be said for evaluation instruments’ [34]. None of these tools addressed all dimensions of IPC compliance as defined by the US Health Care Infection Control Practices Advisory Committee [35]. The most common elements of IPC emphasized in compliance-measuring tools were PPE, HH and sharps safety. Observational studies designed to assess compliance frequently have methodical bias and poor design. A 2019 review by Jeanes *et al*. assessed 71 publications which measured HH compliance; they found that none of the studies were free of bias, and concluded that HH compliance measured by direct observation lacked validity [36]. The act of observation itself changes outcomes (the Hawthorne effect). The extent to which HH compliance changes when HCWs are aware they are being observed was examined by Purssell *et al*. in 2020. Their systematic review and meta-analysis found that the Hawthorne effect varied in scale from -6.9% to 65.3% [37]. Methodological issues were identified in all forms of observation of HH compliance. The existing gold standard, direct observation, was found to likely yield an inflated sense of adherence and provide false reassurance.

### Education and knowledge

Educational interventions are a major focus of effects to improve IPC compliance. However, paradoxically, HCWs with a high level of knowledge about HAIs had lower rates of IPC compliance than those with less understanding of HAIs [21,38]. Gur-Arie *et al*. showed that when HCWs feel particularly threatened by a certain disease, they are more likely to adhere to IPC [39]. Emergent disease outbreaks, such as COVID-19, offer a strong example. In a 2021 survey of 1757 HCWs in Qatar, Abed Alah *et al*. showed that self-reported IPC compliance increased significantly during the COVID-19 pandemic. Median self-reported compliance scores before and during the emergence of COVID-19 increased from 7/10 to 9/10 for use of PPE, and from 8/10 to 9/10 for HH, with *P*<0.001 and large effect sizes (*r* = 0.87 and 0.89), respectively [6].

### Safety culture

Safety culture as an organizational ethos is a predictor of IPC compliance [7], and other workplace culture factors may also influence compliance. A 2012 cross-sectional survey found that ‘higher staff engagement was associated with greater knowledge scores, better hand hygiene practices, fewer reported barriers, and more positive attitudes’ [40]. The inverse is also true. When IPC are not supported by a culture of safety, some staff view the infection prevention behaviours of others as a cynical performance designed ‘to give the impression that infection prevention and transmission were taken seriously’ [41].

## Discussion

The burdens of complying with IPC for HCWs can be mapped across multiple domains. The main findings were general bodily discomfort, with specific emphasis on dermatological, sensory and neurological symptoms. Personal freedoms are also affected, with IPC limiting hand adornment and conflicting with religious commitments. Additionally, compliance requires time, adding pressure to staff who are already time-poor.

This scoping review identified several burdens that have been reported in the literature, but there are other *potential* impacts of IPC which have not been well studied to date. These may provide useful avenues for future research. These potential burdens include effects on: (i) freedom of association and movement, implemented as part of IPC interventions including quarantine or self-isolation or changes to break routines; (ii) free choice of occupation, which may be limited by IPC that prevent HCWs from working at and/or travelling between sites during an epidemic [42]; (iii) privacy, resulting from requirements that HCWs disclose (infectious) health conditions, undergo screening and receive treatment for specific infectious diseases [43]; and (iv) free choice regarding medical care, which may be limited by IPC requiring screening of staff for infectious diseases and/or decolonization for certain pathogens including antibiotic-resistant *Staphylococcus aureus* [44].

Designing IPC to minimize burdens for HCWs may have several advantages, including improved compliance, job satisfaction and staff retention. Healthcare institutions arguably have a reciprocal obligation to staff, not only to provide the means necessary for appropriate IPC (e.g. PPE supplies) but also to minimize unnecessary burdens [45]. One way to do so may be to focus IPC on the most effective interventions, and reduce burdensome IPC with minimal benefits. Further research may be needed to study individual interventions, especially where these were implemented as part of a package of interventions, meaning that the effectiveness of components of such IPC may not have been measured.

This review found that IPC create burdens for HCWs in addition to their benefits for HCWs and patients. While the evidence base regarding the benefits of some measures such as HH is relatively robust, the same cannot be said of all IPC interventions. Indeed, only recommendations relating to HH (the World Health Organization’s Five Moments of HH, use of alcohol-based hand wash, and use of soap and water when hands are visibly soiled) and airborne precautions in the setting of known respiratory infection are *strong recommendations* in IPC in Australia [44]. Strong recommendations are made when the National Health and Medical Research Council is ‘Confident that the desirable effects of adherence to a recommendation outweigh the undesirable effects. Overall the recommendation is based on high quality evidence and is strongly recommended for implementation’. Historically, the burdens for HCWs have been neglected by researchers. While there is an emerging body of evidence in this area, much of this evidence, like the evidence for the benefits of some IPC, is of low quality. As effective IPC is key to protecting patients from infection, and as burdens for HCWs may undermine compliance, there is an urgent need for higher quality (e.g. randomized or quasi-experimental) studies assessing both the benefits and burdens of IPC to refine current policy and practice. Compliance and outcomes could be improved by identifying which measures are the most beneficial, and opportunities to redesign interventions to minimize burdens where possible.

### Limitations

Existent literature largely focuses on barriers to compliance, rather than the burden of compliance. Consequently, the search strategy often relied on identification of burdens implicit in descriptions of barriers. Further research, including qualitative research with HCWs, may help to clarify what HCWs see as the most important burdens related to IPC compliance. This study examined burdens for HCWs, but burdens of IPC for patients may also be important.

The COVID-19 pandemic drew significant attention to the importance, and associated burdens, of compliance with PPE. The large body of literature that has emerged in the post-pandemic context potentially weakens this analysis, as minimal distinction has been drawn between pre- and post-pandemic sources.

### Conclusion

In conclusion, more than three-quarters of HCWs will experience adverse events related to the use of PPE [21]. Given that other IPC may also be burdensome, these issues affect the vast majority of HCWs. Documented harms include those to the physical bodies of HCWs, their autonomy and their relationships with work. Perhaps unsurprisingly, compliance with IPC appears low. In seeking to boost compliance and prevent HAIs, healthcare institutions should not disregard the lived experiences of those tasked with its implementation, and arguably have a reciprocal obligation to develop IPC which can be implemented with minimal hardship. Further research should clarify the burdens experienced by HCWs, and quantify the benefits of individual IPC in order to select the measures with the greatest benefits and lowest burdens for staff and patients.

## Supplementary Material

Supplementary data to this article can be found online at https://doi.org/10.1016/j.jhin.2023.12.003.

Supplementary Material
