# Supplementary material for: Burdens of infection control on healthcare workers: a scoping review
Source: J Hosp Infect. Author manuscript; Available in PMC 2025 Jul 5. (PMC7617856; doi:10.1016/j.jhin.2023.12.003)
Supplement: Supplementary Material [file EMS206538-supplement-Supplementary_Material.docx]

**Appendix 1**

Search terms

The following free-text terms were used in addition to the MeSH terms described below: ‘Burdens’ and ‘Compliance’

| MeSH term | MeSH unique ID | Entry terms |
| --- | --- | --- |
| Infection Control | D017053 | - Control, Infection |
| Universal Precautions | D016635 | - Precautions, Universal - Precaution, Universal - Universal Precaution |
| Hospital Personnel | D010564 | - Hospital Personnel |
| Health Personnel | D006282 | - Personnel, Health - Health Care Providers - Health Care Provider - Provider, Health Care - Healthcare Providers - Healthcare Provider - Provider, Healthcare - Healthcare Workers - Healthcare Worker - Health Care Professionals - Health Care Professional - Professional, Health Care |
| Guideline Adherence | D019983 | - Adherence, Guideline - Policy Compliance - Compliance, Policy - Protocol Compliance - Compliance, Protocol - Institutional Adherence - Adherence, Institutional |
| Health Knowledge, Attitudes, Practice | D007722 | - Knowledge, Attitudes, Practice |
| Quality of Healthcare | D011787 | - Health Care Quality - Quality of Healthcare - Healthcare Quality - Quality of Care - Care Quality - Pharmacy Audit - Audit, Pharmacy - Pharmacy Audits |

## **Appendix 2**

## Results and source descriptions

| First author | Title | Year | Country | Study type | Theme | Key findings |
| --- | --- | --- | --- | --- | --- | --- |
| AbedAlah | Compliance and barriers to the use of infection prevention and control measures among health care workers during COVID‐19 pandemic in Qatar: a national survey | 2021 | Qatar | Retrospective observational study (survey) | COVID-19 | Self-reported PPE use and HH increased significantly during COVID-19. Training did not improve compliance |
| Alhumaid | Knowledge of infection prevention and control among healthcare workers and factors influencing compliance: a systematic review | 2021 | Saudi Arabia | Systematic review and meta-analysis | Knowledge and compliance | Predictors for non-compliance: time constraints, higher patient-to-nurse ratio, and professional specific  Barriers to compliance: lack of supplies and adverse reactions to HH products |
| Arrowsmith | Removal of nail polish and finger rings to prevent surgical infection | 2014 | UK | Systematic review and meta-analysis | Burden of compliance | No trials have investigated whether wearing nail polish or finger rings affect the rate of surgical wound infection |
| Atashi | Health-care workers' experience of stressors and adaptation strategies for COVID-19: a qualitative research | 2022 | Iran | Qualitative interviews | COVID-19 | Stressors related to PPE and COVID-19: reduced concentration, disruption of normal diets and rest, PPE shortages, lack of protocol for infected workers |
| Blenkharn | Rigid infection prevention and control rules and religious discrimination: an uncomfortable juxtaposition? | 2020 | UK | Review of legal cases | Discrimination | Generic policies impose an asymmetrical burden on HCWs |
| Brooks | Factors affecting healthcare workers' compliance with social and behavioural infection control measures during emerging infectious disease outbreaks: rapid evidence review | 2021 | UK | Rapid systematic review | Factors affecting compliance | Physical harms due to PPE: dehydration, skin peeling, difficulty breathing, sweating and dizziness, headaches and skin rashes  Staff who were comfortable wearing protective eyewear and N95 respirators were significantly more likely to wear them |
| Cimon | Jewellery and nail polish worn by health care workers and the risk of infection transmission: a review of clinical evidence and guidelines | 2017 | Canada | Review | Burden of compliance | Rings in a surgical setting did not result in an increased risk of surgical site infections |
| de Oliveira | Factors related to poor adherence to hand hygiene in  healthcare delivery: a reflection | 2014 | USA | Discussion paper | Factors affecting compliance | Barriers included a lack of supplies; skin irritation; cultural expectations; personal motivation; workplace culture; training |
| Desai | COVID-19 and personal protective equipment: treatment and prevention of skin conditions related to the occupational use of personal protective equipment | 2020 | USA | Research letter | Burden of compliance | Skin conditions arising from mask use: contact and moisture-associated dermatitis, pressure-related skin injury, and acne |
| Fix | Health care workers' perceptions and reported use of respiratory protective equipment: a qualitative analysis | 2019 | USA | Observational study (qualitative interviews) | Burden of compliance | Respiratory protective equipment was described as ‘suffocating’ and ‘claustrophobic’ |
| Galanis | Healthcare professionals’ knowledge and practices towards hospital infections in surgical clinics | 2021 | Greece | Retrospective observational study (survey) | Level of knowledge | Overall knowledge was low |
| Galanis | Impact of personal protective equipment use on health care workers' physical health during the COVID-19 pandemic: a systematic review and meta-analysis | 2021 | Greece | Systematic review and meta-analysis | Burden of compliance | The estimated overall prevalence of adverse events among HCWs was 78% (95% CI 66.7–87.5%) |
| Houghton | Barriers and facilitators to healthcare workers' adherence with infection prevention and control (IPC) guidelines for respiratory infectious diseases: a rapid qualitative evidence synthesis | 2020 | Ireland | Review | Barriers to compliance | The discomfort of wearing PPE and increased workload arising from IPC were barriers to adherence |
| Jain | Factors limiting the usage of personal protective equipment in a tertiary-care hospital | 2013 | India | Retrospective observational study (survey) | Knowledge and compliance | Compliance ranged from 36% in low-risk settings to 58.6% in high-risk settings (*P*=0.016) |
| Madan | Barrier precautions in trauma: is knowledge enough? | 2002 | USA | Retrospective observational study (survey) | Knowledge and compliance | Self-reported rates of compliance provided an overestimate for use of all components of contact precautions except gloves (*P*<0.02) |
| McArdle | How much time is needed for hand hygiene in intensive care? A prospective trained observer study of rates of contact between healthcare workers and intensive care patients | 2006 | UK | Prospective observational study (survey) | Burden of compliance | Post-contact HH rates were 43% for direct contacts and 12% for indirect contacts  100% compliance by all healthcare workers would require about 230 min/patient/day |
| McAteer | Using psychological theory to understand the challenges facing staff delivering a ward-led intervention to increase hand hygiene behavior: a qualitative study | 2014 | UK | Observational study (qualitative interviews) | Barriers to compliance | Barriers to IPC: lack of time, staffing levels, perceptions of impracticality, negative perception from other staff, stress, organizational and motivation |
| McGuckin | Irritant contact dermatitis on hands | 2017 | USA | Review | Barriers to compliance | Handwashing, alcohol rub and gloves are associated with higher rates of skin irritation. Irritated skin harbours a higher bacterial load than non-irritated/intact skin |
| Parush | Human factor considerations in using personal protective equipment in the COVID-19 pandemic context: binational survey study | 2020 | Israel and Portugal | Retrospective observational study (survey) | Barriers to compliance | There is a relationship between the difficulties in hearing and seeing, created by PPE, the discomfort of wearing PPE, and reduced situational awareness |
| Sharma | Barriers faced by health-care workers in use of personal protective equipment during COVID pandemic at tertiary care hospital Uttarakhand, India: a qualitative study | 2022 | India | Observational study (qualitative interviews) | Barriers to compliance | Fatigue, reduced oral intake, headache, discomfort, limited privacy |
| Srinath | Headache of wearing PPE; a survey for neurological symptoms with PPE amongst health care workers’ | 2022 | India | Retrospective observational study (survey) | Burden of compliance | 44.98% HCWs developed headache after donning PPE, with median intensity peaking at 6/10 (visual analogue scale) |
| Valim | Instruments for evaluating compliance with infection control practices and factors that affect it: an integrative review | 2014 | Brazil | Review | Measuring compliance | None of the instruments identified addressed dimensions of IPC compliance as defined by the Centers for Disease Control and Prevention |
| Visscher | Hand hygiene compliance and irritant dermatitis: a juxtaposition of healthcare issues | 2012 | USA | Review | Hand hygiene | Incidence of dermatitis: up to 85% of nurses had histories and 25% reported dermatitis symptoms |
| Ward | Hand adornment and infection control | 2007 | UK | Summary of expert opinion | Hand hygiene | Published research seems to back up general recommendations regarding artificial nails and jewellery on the hands, but evidence against nail polish is conflicting |
| Weber | Occupational health risks associated with the use of germicides in health care | 2016 | USA | Clinical audit | Barriers to compliance | Injury due to chemical exposure was rare. Limited data link dermatitis and eczema in HCWs to low-level disinfectants |

COVID-19, coronavirus disease 2019; PPE, personal protective equipment; HH, hand hygiene; IPC, infection prevention and control; HCW, healthcare worker; CI, confidence interval.
